# Self-experienced sexual and reproductive health in young women with Attention Deficit Hyperactivity Disorder a qualitative interview study

**DOI:** 10.1186/s12905-022-01867-y

**Published:** 2022-07-14

**Authors:** Karin Wallin, Inger Wallin Lundell, Lena Hanberger, Siw Alehagen, Sally Hultsjö

**Affiliations:** 1 Department of Obstetrics and Gynecology in Linköping, Department of Health, Medicine and Caring Sciences, Linköping University, Linköping, Sweden; 2 Department of Health Sciences, The Swedish Red Cross University, Huddinge, Sweden; 3 Department of Health, Medicine and Caring Sciences, Linköping University, Linköping, Sweden; 4 Department of Psychiatry, Ryhov County Hospital, Jönköping, Sweden

**Keywords:** Attention Deficit Hyperactivity Disorder, Health promotion, Reflective thematic analysis, Sexual and reproductive health, Young women

## Abstract

**Background:**

Sexual risk behaviors and struggles in romantic relationships result in higher risk of unplanned pregnancy, sexually transmitted diseases, sexual victimization and lower satisfaction in relationships for young women with Attention Deficit Hyperactivity Disorder (ADHD). There is a need to better understand sexual behaviors and the consequences of relational difficulties to help health professionals promote sexual and reproductive health. To deepen knowledge in this area, this study aimed to identify and describe self-experienced sexual and reproductive health in young women with ADHD.

**Methods:**

A qualitative design was used. Data was collected with individual and focus group interviews with 15 young women, aged 15–29, with an ADHD diagnosis, and analyzed with thematic analysis.

**Results:**

Data analysis identified the themes *Acceptance of being different* and *Feeling sexually secure.* The women reveal feelings of being different from others without ADHD as they break norms of sexual behavior, struggle with romantic relationships, and have difficulties concentrating during sex. There is a need to be understood and accepted, to not feel judged, and to manage romantic relationships. Self-knowledge helps them to recognize needs for support and to develop strategies that can improve sexual satisfaction. Feeling sexually secure illustrates the women’s need to feel comfortable with their own sexuality and in control in the sexual situation. Low self-esteem and a negative self-image, described as a consequence of living with ADHD, can compromise communication in sexual situations and increase fear of being rejected. Further, misjudging sexual partners and situations can contribute to sexual victimization.

**Conclusions:**

This study provides knowledge of how ADHD affects emotions and sexual behaviors in young women. The results highlight the need for understanding and acceptance by peers and partners. It accentuates the value of involving the partner in counselling and the importance of self-knowledge. Feeling insecure in sexual relationships further implies the importance of early diagnosis to prevent secondary outcomes of ADHD, and the need for sexual victimization screening in professional settings.

## Background

In young adulthood, sexuality is explored through sexual experiences with oneself and others. Positive sexual experiences are seen as a source of emotional, mental, and social well-being and can provide communication skills and self-esteem, essential for well-functioning relationships in the future [1–3]. For young women with Attention Deficit Hyperactivity Disorder (ADHD), struggles with social interaction, self-regulation and mental health could all risk affecting sexual experiences and sexual and reproductive health negatively. Sexual risk-taking behaviors and struggles with close romantic relationships result in a higher likelihood for unplanned pregnancy, sexually transmitted diseases, sexual victimization, and lower satisfaction with relationships than is found in young adults without a diagnosis [4–7].

ADHD is a worldwide neurodevelopmental condition. According to the Diagnostic and statistical manual of mental disorders (DSM-5) ADHD is characterized by symptoms of inattention and/or hyperactivity and impulsiveness with impairment of daily function. ADHD is categorized into three types depending on prominent symptoms; predominantly hyperactive and impulsive, predominantly inattentive, and a combined type [8]. Most symptoms persist into adulthood. Two larger studies concluded adult prevalence to be 2.8% and 5% respectively in multinational samples, where women represented approximately 40% [9, 10]. Comorbid disorders such as Autism Spectrum Disorder (ASD), personality disorders and substance use disorders are common [11, 12]. Women with ADHD show higher rates of anxiety disorders and depression as well as self-harm behaviors compared to men with ADHD and women without a diagnosis [13–16].

Symptoms of ADHD are associated with sexual risk-taking in young adults as lower age of sexual initiation and more sexual partners than young adults without ADHD resulting in a higher risk of contracting a sexually transmitted disease (STD), having an unplanned pregnancy or becoming a parent at a young age [4, 7, 17–20]. Sexual risk-taking in young adults with ADHD has been shown to be mediated by delinquency and substance use [19, 21]. Young women with ADHD seem to have similar risk-taking behaviors as young men with ADHD; however, studies with only female participants are rare. In an all-female sample Hosain et al. [22] revealed how women with ADHD symptoms used alcohol more often before they had sex and were more likely to be involved with a risk-taking partner than women without symptoms while Huggins et al. [23] found that they were the least likely to use condoms compared to both men with ADHD and women without a diagnosis. Sexual risk-taking in women with ADHD is also linked to sexual victimization, which can be explained by the increased risk of meeting a partner with an unknown sexual history when repeatedly engaging in casual sex [6]. Even though women are generally more prone to sexual victimization then men, Snyder [24] suggested that being inattentive and not always perceiving the risks made women with ADHD especially vulnerable for exploitation by an offender.

Despite having several partners, young adults with ADHD struggle to handle close romantic relationships and report lower satisfaction in relationships and sexual life than adults without a diagnosis [25–27]. Among college students, ADHD symptoms indicate higher perceived fear about forming close relationships where previous negative experiences influence future fears and lower expectations of intimacy in the relationship [28]. Young women with ADHD also report fewer romantic relationships compared to women without ADHD [29] as well as poor relational quality, suggesting difficulties with hostile conflict resolution and poor emotion regulation [5].

Within the research field of ADHD, there is still a lack of studies including women [30]. Sexual risk behaviors in young men with ADHD have been well explored in recent decades and even though later research has become more gender diverse, there is still a need to better understand the motivations and sexual behaviors in young women with ADHD [21, 31, 32]. Research also needs to explore the underlying reasons of low satisfaction in romantic relations and sexual life as well as fear of close relationships since well-functioning close relations are important for overall well-being and sexual satisfaction [3, 33].

Previous studies in sexual and reproductive health, including young women with ADHD, have mainly used self-reported questionnaires [5, 23, 28] population-based registries [7, 18] or structured interviews [22]. This study intends to deepen the understanding of the women’s own experience of sexual and reproductive health by using a qualitative approach. The result could contribute to better understanding of the women’s challenges and needs in sexual situations and romantic relationships, proving useful knowledge for health professionals in fields of psychiatry and women’s health to individualize counseling. Deeper knowledge could help health professionals to support women to identify strategies, and increase perceived self-competence that promotes healthy sexual behaviors [34].

Thus, the aim was to identify and describe self-experienced sexual and reproductive health in young women with ADHD.

## Methods

### Design

A qualitative design was adopted, which according to Patton [35] is appropriate as it allows new insights. Data was analyzed with reflexive thematic analysis [36, 37], adopting a constructionist stance; hence, knowledge was assumed to be situated in the women’s context, and depended on the researchers’ subjective interpretation of data [37, 38].

### Recruitment and participants

Women with an ADHD diagnosis aged 15–29 were approached for participation at two psychiatric outpatient clinics, two youth guidance centers in public health care and through Facebook pages that belonged to three different interest groups for people with ADHD. The youth guidance centers were health clinics specializing in sexual and reproductive health, available to adolescents and young adults without referral. All four clinics were situated in different cities in the south-east and middle region of Sweden including medium-sized and large cities. Psychiatric clinics were run by one university hospital and one regional hospital. In the psychiatric clinics, women with a recorded ADHD diagnosis given by a physician or psychologist, were asked to participate. Due to respect for personal confidentiality, youth guidance centers cannot see recorded psychiatric diagnoses and they could therefore not be confirmed. Women that self-reported an ADHD diagnosis, when asked for their medical background, were therefore informed about the study. To better ensure the accuracy of the ADHD diagnosis, for women recruited from youth guidance clinics and Facebook pages, questions were asked about when and where the diagnosis was set and by whom. Written information was given by health professionals in connection with a clinical visit, or via Facebook pages that belonged to the interest groups. Interested women were asked to leave their phone number for additional verbal information or talked to the first author when available at one of the clinics. In total, 18 women consented to participation, and 15 participated in interviews. Background characteristics of the participating women are presented in Table 1. To provide a safe environment where the women could feel free to speak of their experiences, the time and place for interviews was chosen by the women. A purposeful sampling was used, which strived for variation and information-rich cases to allow study the women’s experience in depth [35]. Recruitment took place between December 2019 and January 2021. The recruitment process was paused between March 2020 and August 2020 due to Covid-19.

**Table 1:** Characteristics of participants

Variable	Participants (n = 15)
Age	25 (15–29)*
Age at time of diagnosis	24 (7–29)*
*Level of education*	
Elementary school	4
Secondary school	9
University	2
*Employment*	
Full time, work	3
Part time, work	3
Unemployed	1
Student, elementary school	1
Student secondary school	2
Student, university	4
Parental leave	1
*Relational status*	
Living with a partner	4
In a relationship, not living with a partner	3
Single	8
*Neuropsychiatric diagnosis*	
ADHD- combined type	9 (4)**
ADHD- predominantly attentive type	2
ADHD- combined type and ASD	4
*Psychiatric diagnoses, current or lifetime history of*	
Depression	5 (2)**
Anxiety	9 (4)**
Obsessive compulsive disorder	1
Eating disorder	2

*Median (range); **Number (number of self-reported)

### Data collection

Data was collected in 12 individual interviews and one focus group interview including three participants. The interview was semi-structured. Questions was flexible in order, but the interviews started with the same question (Table 2).

**Table 2:** Table of questions

Starting question	Can you tell me what sexual health means to you?
Sexuality	Can you tell me something about your sexuality that you appreciate?
Relationships	How do you view your sexual relationships?
	What are your challenges? Strengths?
	What are important for you to make causal/romantic relationships work?
Sexual situations	Can you tell me about a sexual situation with yourself or with someone else that felt good/did not feel right? What contributed? What could have helped you to make it better?
ADHD	Do you think ADHD can affect your sexual and reproductive health? In what ways?
Needs	What do you need to experience sexual and reproductive health? Who do you turn to for support?

The first question “Can you tell me something about your sexuality that you appreciate?” was not effective to encourage the women in the focus group and first individual interview to freely start speaking about their experience and was therefore modified to “Can you tell me what sexual health means to you?” To reach a deeper understanding and to clarify statements, questions such as “Can you tell me more? and “Can you explain?” were used. The focus group and first individual interview were conducted to test the semi-structured questions. The questions were found to answer the aim of the study and the interviews resulted in rich data. Except for modifying the starting question, the questions were not changed, and the interviews were included in the analysis.

The focus group interview was conducted online via Zoom, using the written chat forum that previously had been shown to be a valid method for discussing sensitive subjects such as sexual health [39]. The video feature was not used, and participants could choose to be anonymous to each other. Only the invited participants could log in. The written text of the chat was automatically downloaded to a computer, and it was not possible to log in after the group session was finished. The first author acted as moderator and the last author, with experience of focus group interviews, as an assessor. The intention of the study was to conduct several focus groups. Due to difficulties in remembering time, not feeling emotionally well, and worries about discussing an intimate subject with others, some participants did not show up therefore only individual interviews continued to be performed. Several women expressed a wish to still be a part of the study and were offered the opportunity to participate in individual interviews. All but one interview was performed by phone. Interviews were digitally recorded and lasted 30–80 min (median 55 min). Interviews were performed and transcribed verbatim by the first author. Transcripts were double checked with the recordings to ensure accuracy. Initial notes were made after the interviews and during transcription that reflected the data collection session and ideas of interest for analysis. Reflecting on data it was concluded that interviews were rich in data and did not yield any new information. No more interviews were therefore conducted.

### Data analysis

Reflexive thematic analysis was used to analyze the data following Braun & Clarkes’ six steps of analysis. The method makes it possible to find patterns in data and deepens the understanding by identifying underlying ideas and assumptions [36]. The first author led the data analysis, but all steps were continuously discussed within the whole research team. First the research team familiarized themselves with the data by reading and rereading the transcripts as well as previous written notes of interest. While reading, impressions and ideas of meanings and patterns were written down. All interesting features the answered the aim in the data set were coded. The themes were revised by checking codes for coherent patterns within themes, ensuring clear distinctions between themes and verifying that those themes reflected the whole data set. All authors were involved in next step of once more refining the themes, identifying the essence of the themes, and constructing informative names. The final patterns of themes and subthemes and their meaning are presented in the results, illustrated by quotes.

The research analysis was guided by the “15-point checklist for good thematic analysis” by Braun and Clarke [36], ensuring rigor in the data analysis. For example, all data was given equal attention to make sure it was not generated from a few examples, and all codes were sorted into potential themes. The themes were checked against each other and against the data set. All data was analyzed and interpreted to make sense of it. In line with the method, the research team went back and forth in the analysis process, and constantly reflected on the assumptions made in the interpretation. Disagreement was resolved by discussion rather than by seeking consensus on meaning, which resulted in a more nuanced reading of the data [37]. Research team members had knowledge and experience of the used methodology, and were all active in the data analysis, which enhanced the credibility of the study, helping to confirm that the women’s views fitted the presentation of the study [40]. The research team also had knowledge and working experience in the fields of sexual and reproductive health, psychiatric care, or child- and adolescent care. Even though researcher subjectivity was seen as a resource in the research process, it was necessary to reflect on previous knowledge and experiences to prevent over-interpretation of data [36]. The trustworthiness of the study was strengthened by the fact that the study followed the criteria for reporting qualitative research (COREQ) [41].

## Results

**Fig. 1:**
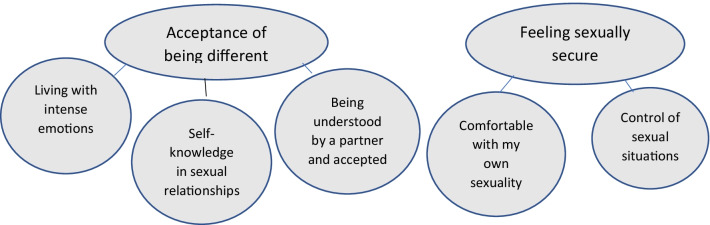
Themes and subthemes—self-experienced sexual and reproductive health in young women with ADHD

The data analysis resulted in two themes with attached subthemes, presented in Fig. 1, and these are further described in the text below.

### Acceptance of being different

ADHD is perceived as an integrated part of the women that makes them different from others. Emotions and difficulties focusing affect how they experience and act in casual sexual relationships as well as romantic relationships, sometimes breaking norms of sexual and relational behaviors. The women feel the need for peers and partners to understand their emotions and behaviors and to accept them for who they are. Lack of acceptance is described as leading to feelings of guilt and shame, and difficulties in maintaining relationships. Self-knowledge of emotions and behaviors helps them to accept themselves and to adopt useful strategies in sexual relationships.

#### Living with intense emotions

The women find the experience of emotions to be more intense for them than for people without ADHD. When meeting others, strong feelings of desire and attraction can arise suddenly and may trigger the impulse to engage in temporary sexual relationships.

There are a lot of impulses and a lot of, now I’m horny and then I should have sex, and then you might not think about, well you shouldn’t have sex with anyone sort of. Woman 10.

Even though one-night stands have resulted in unplanned pregnancy, abortion, STD and conflicts in relationships, several women also see them as something exciting and fun. Some describe causal sex to be a way of letting go of extra energy, helping them to concentrate on daily life. The women describe how friends and family do not always understand the positive sides of their behavior. Instead, lack of acceptance for their actions has led to feelings of shame. Not following accepted norms of sexual behavior of young women is experienced as making acceptance from those around them more difficult. Especially at a young age, several women describe being judged by peers and called “slutty” or “easy to get into bed with”.

But otherwise, I have always felt well. But I have felt bad about the reaction that I received from my environment. Uhm, I have felt myself…that I have been judged sort of. It is more than that, I have felt shame sort of after sex but not because of the sex itself but from the way I have been treated afterwards by the others when I tell them. Woman 6.

To engage in sexual behaviors that are not seen as acceptable is also experienced as making it more difficult for the women to ask for help from friends or health professionals. The women describe the shame in going to the health clinic to repeatedly test for STD or revealing self-harming behaviors to deal with anxiety such as having sex with everyone who wants to or always looking for dangerous situations. However, their experience when seeking health care is mostly perceived as positive.

“ I test myself a lot because I am usually not with the same partner for particularly long, and I used to worry a lot that the staff would judge me but I really don’t think they do” Woman 2.

#### Self-knowledge in sexual relationships

Gaining self-knowledge has for some women been an important step in accepting who they are, and which has made it easier to acknowledge their strengths and weaknesses and develop strategies that can help them. Self-knowledge, along with ADHD medication and life experience, have helped them to recognize their behaviors and avoid risky sexual situations. Several women highlight the importance of receiving an ADHD diagnosis to better understand why they feel and act the way they do. Knowledge of why sexual relationships are more difficult for them than others has helped some women to accept who they are, and some have found it easier to see the positive sides of themselves e.g., being outgoing and open-minded and not judging others.

“Having ADHD…I know I have come very far and have accepted the way I am. I know myself and can focus on making it as good as possible. But I can still feel sometimes…like I’ve been too much, or said too much, in ways I regret. But I have become more permissive with myself, and I reach out to friends that can confirm that I am alright” Woman 7.

Acknowledging and accepting their difficulties has made it easier for the women to develop strategies that improve concentration in sexual situations. They describe difficulties focusing when having sex, both with themselves and with a partner, influencing the sexual satisfaction negatively. Even when they want to have sex it can be difficult to stay concentrated for a long period of time. The mind easily wanders away, distracted by something in the room, or because of constant thoughts in the head or lack of variation during sex. Being aware of their challenges to stay focused and to keep sexually aroused, some women have adopted strategies such as: changing positions often, putting on music or TV, decorating the room to reduce stimuli, taking ADHD medication before sex or asking the partner to be attentive to them. Strategies are found easier to adapt in longer relationships where the women can communicate their difficulties to a partner.

I quickly lose focus and unfortunately, I do it in sexual situations as well. Uhm..so it can’t be too long-winded. Something must happen, change a position or something of that sort of because otherwise I can’t keep myself focused. It doesn’t matter how much I want to. So that is also something we have had to learn. But then again communication becomes very important. Because it’s not like I get tired of him or I don’t want to be with him. Woman 4.

#### Being understood by a partner and accepted

When meeting new people, several women adjust their behavior to fit in, describing themselves to be difficult or too much. This works in temporary sexual relationships but not in the long run. To build a romantic relationship, they need the partner to understand how the diagnosis affects them and to appreciate and accept them for who they are.

“I restrain myself because I know which behaviors are not acceptable. But it becomes an effort to not be myself fully […] and you can’t do that forever if you’re seeing someone” Women 11.

To establish a romantic relationship the women, express the need for the partner to understand underlying reasons for their initial emotions. At the beginning of a relationship the women describe overwhelming strong feelings. They show a lot of engagement in the relationship, sometimes leading to disappointment when the partner does not put as much effort into it as they do. Some experience becoming easily bored with the relationship and their sex life when the initial desire and attraction declines.

“I didn’t understand why I couldn’t find anyone. How can I possibly find someone that can handle me? I’m not shaped in ways that others are, I don’t follow the same patterns. […] But my demands are higher now. I need someone who is more like me, who tries to understand the underlying causes of my feelings, not giving up too early. Someone who accepts me for who I am…and doesn’t expect too much.” Women 9

To manage an established relationship, it is important that the partner is aware of sudden changes in mood and the fact that it can be difficult for the women to sometimes take part in activities even when they want to, it can result in partner support and avoiding conflict. When the women feel free to be who they are they find it easier to bond emotionally and sexually with the partner.

The women find it helpful that the partner understands how sexual desires are affected by symptoms of ADHD and psychological well-being. Several women state that general stress, depression, anxiety, premenstrual syndrome and vestibulitis have a negative impact on sexual desire. While some women experience that ADHD medication and hormonal contraception make them feel low and not interested in sex, others find medication for depression to directly influence their sexual desire. However, medication for ADHD and depression has given them better well-being overall, making them more interested in sex and more likely to feel desire. Those women who have partners that accept that the women do not always feel like having sex, and understand why, do not see it as something that compromises the relationship.

“It affected me very much in the beginning. Because he first thought that it was something between him and me, that he did something wrong or something. Uhm, but then I explained that you are not the problem, that I don’t feel well and I can’t pretend that everything is well because it’s not. Uhm, and the only thing you need to do is to be there and hold me and not to push me to do anything.” Woman 10.

### Feeling sexually secure

To have pleasurable sexual experiences the women express a need to feel sexually secure. When the women feel comfortable with their sexuality and have control of the sexual situation, sexual experiences can become a source of joy and affect personal well-being as well as strengthening romantic relationships. Feeling insecure in a sexual relationship or situation can have opposite effects.

#### Comfortable with my own sexuality

The women describe feeling comfortable with sexuality as knowing what they like in a sexual situation and being able to communicate feelings, desires, and expectations to a partner without being ashamed or afraid.

“Woman 1: To feel comfortable with one’s sexuality is in my opinion to know what you want, not to feel insecure and not to be ashamed about it.

Woman 3: I agree, it’s about feeling secure and knowing what you want.”

They express how feeling comfortable benefits sexual enjoyment on equal terms and creates feelings of intimacy. In turn, the women find feelings of intimacy, such as trust and closeness, to make them more relaxed with a partner in a sexual situation, creating a feeling of sexual security. Feeling comfortable with sexuality is revealed to be closely influenced by how the women view and value themselves.

Several women are struggling to feel comfortable with sexuality because of negative self-image and low self-esteem, described as a result of living with ADHD. Perceived failure, not only in sexual relationships but throughout life, e.g. in school or work achievements and friend relationships, is acknowledged to affect how they act and experience sexual situations. Some are afraid to make wrong decisions that could disturb the sexual excitement or hurt the partner’s feelings, meaning they focus on their partner’s wishes during sex instead of communicating their own.

Well I guess it is just to say that it hurts or that wasn’t so comfortable. Oh it is difficult to say why it has been difficult for me to say it. Uhm, really, but it, but I guess it’s about not ruining the mood, maybe. Uhm, and again that you don’t think that it is so important, well he, I guess he can go on. Well he thinks it’s nice and that you, so to speak, don’t really make the connection of sex being for both sakes, for both pleasure. Woman 5.

Experiences of being perceptive to other people’s feelings due to ADHD can also result in insecurities during sex for the women, as they easily interpret a word or a look as something negative, making them question their body or their performance.

Even though some of the women describe themselves as being sexually confident, enabling them to enjoy and act freely in one-night stands, they doubt they could manage a romantic relationship. Some experience feelings of only being good enough for sex, and they fear being rejected by a partner if the woman or the partner did not feel like having sex. Previous experiences of early rejection in romantic relationships have also made it difficult to be sure of the partner’s intentions. Women that stay in romantic relationships describe the importance of affectionate touching and small gestures of appreciation. It gives them feelings of intimacy that assure them that the partner really wants to be with them.

...that he will only be with me for the sexual part if I don’t give it to him he will leave me. It was the way I saw it and it scared me sort of. (…) And that was probably about insecurity, that maybe I didn’t have enough value be allowed to stay, no matter what. Uhm…and I guess that’s why it has become like this, to know that we are always on the same level. It’s not only about sex, it is about us being in a relationship and in a relationship it is important there is touching. Women 3

Being comfortable with sexuality is experienced by the women to become easier with age and experience. Early sexual experiences are influenced by expectations from surrounding peers and not by one’s own wishes, leading to feelings of insecurity in the sexual situation. Several women say that they had penetrative heterosexual sex at the age of 14–16 because it was expected of them. Some women have sex with men because it is the perceived norm, even though they are attracted to other women. It is found difficult to communicate their feelings not knowing what is normal and because sex focuses on the man’s desires. For some women, having sex on other people’s terms has led to difficulties knowing their own needs and wishes as young adults.

To be secure in sexual situations, several women express the wish to explore sex by themselves to learn more about their own bodies and desires before having sex with someone else. Talking freely about sex with other girls was found difficult at a young age, but several of them think that it could have helped them to better understand their own sexuality at that time. Having an older sister or a family member with an ADHD diagnosis to talk to is comforting. Learning from others’ experiences helps them to know what to expect from sex and how and when to set sexual boundaries.

#### Control of sexual situations

The women express a wish to feel in control of the sexual situation, enabling them to relax and enjoy the moment. While having sex by themselves they feel like they are in control when they can ensure privacy in the surrounding environment, checking that they are alone and that they will not be disturbed. With a partner they express the need to be able to say no to a partner in a sexual situation and feel sure that they are listened to. It is found easier to say no to someone they know while being influenced by alcohol or having taken the sexual initiative makes it more difficult.

…when I sort of had a more permanent partner. Sometimes when I... oh well wanted a break, uhm it’s always been ok. It’s almost like I feel more lust afterwards because I felt so secure to know that I could stop if I wanted. Women 7.

Even if the women describe sexual situations as generally safe, several have experienced situations where they felt they were taken advantage of, especially at a young age. Trusting a more experienced partner or saying no after initiating sex has led to unwanted sexual situations where they did not feel listened to. Some women did not fully understand that they were victimized until later. Experiences of sexual victimization have led to feelings of guilt and shame as well as difficulties trusting new partners. Some of them have actively abstained from sexual situations for long periods.

…It felt exciting sort of. I trusted him. (…) we were at his apartment and then it didn’t go the way I thought it would and it was not until a year later I understood it was a rape sort of. I was stuck there for hours and couldn’t leave. It was not about what I needed or wanted, and I learnt that this is the way it’s going to be. It’s just to roll onto your back sort of. (…) …but because of this first experience I had problems trusting men. Woman 9.

## Discussion

The results of this study indicate that being accepted as different and feeling sexually secure could be essential for young women with ADHD to experience sexual and reproductive health.

The women experience feelings of being different from others without ADHD, describing how symptoms affect their actions resulting in repeated casual sex, struggles with romantic relationships, and difficulties concentrating during sex. Understanding and acceptance become important for the women in order not to feel judged and to manage romantic relationships. Self-knowledge can help with self-acceptance and the development of strategies that improve sexual satisfaction. The women also describe the need to feel sexually secure to enjoy sexual relationships and feel safe. They find low self-esteem and a negative self-image to be a result of living with ADHD, making them less comfortable in sexual situations, hindering them from expressing their wishes and desires to a partner, and increasing their fear of being rejected. Being inexperienced and trying to live up to expectations of sexual behavior and misjudging sexual situations further compromise their control of the situation and can lead to unwanted sexual situations.

The theme acceptance of being different describes how the women’s impulsive behavior can lead to unprotected casual sex with unknown partners, which results in unplanned pregnancy, abortion, early parenthood, and STD. These issues could risk having major impacts on their lives due to the increased risk of infertility due to STD [42], limited education and employment associated with early parenthood [43] and the women’s experience of sexual risk-taking to deal with anxiety. But even though the women find consequences of impulsive behavior to have negatively affected them, casual sex is also something that several of them like. This study emphasizes that there is a risk of being judged by others when breaking norms of sexual behavior, coloring the experience with shame and guilt. Health care counseling therefore needs to target contraceptive counseling and questions about reasons for having sex, but also the stigmatization associated with casual sex in young women [44].

The women also describe symptoms of ADHD as complicating the establishment of romantic relationships and problems concentrating during sex. The result of this study strengthens earlier suggestions that concentration problems could be a reason for low sexual satisfaction in women with ADHD [25]. Lack of experience of close relationships among our women, could also lead to a more negative view of sexuality [45], which is suggested to possibly have a negative impact on sexual satisfaction. This study adds to research by illustrating strategies that could improve concentration during sex, e.g., reducing stimuli in the room, taking medicine before having sex, changing positions often and asking the partner to be attentive to the woman. Considering that symptoms of ADHD are associated with impulsive behaviors and challenges in planning ahead [8], adopting the strategies in the moment could be challenging for some of the women. Involving the partner can therefore be helpful for meeting her needs. However, expressing challenges and needs to a partner is found difficult by some of the women in our study. Self-knowledge could be a first step to discovering how to involve the partner. According to Schrevel et al. [46] awareness of weaknesses helped to realize when to ask for help and how to make the partner aware of how they can deal with their behaviors in specific situations. Professional counseling that includes the partner could also facilitate communication in the couple, making it easier for them to adopt strategies focusing during sex and helping the partners to reach an overall understanding of the women’s emotions and actions, described in our study to improve relational quality.

Feeling sexually secure is described in this study to be influenced by secondary outcomes of living with ADHD, such as low self-esteem and a negative self-image. The women in this study reveals difficulties expressing desires and wishes to a partner during sex, stating that they value their partner’s desire higher, putting their own sexual satisfaction aside. They therefore risk missing out on the positive effects that communication can have on sexual and relational satisfaction [47]. In a study of young women without ADHD [48] similar findings of not expressing their own needs in sexual situations were revealed. According to the mentioned study, sexual intercourse for their partners' sake, and the importance of the partner's sexual pleasure can be explained by the influence of gender norms, as the women tries to live up to the perceived ideal to be a woman in a sexual situation instead of valuing their own sexual satisfaction. It is therefore possible that our results could be partly explained by the influence of gender norms. However, the women in our study emphasize that living with ADHD has led to feelings of constant failure in life and a belief that they always make the wrong decisions, with the result that they value themselves less and are afraid of saying something wrong. Early diagnosis and treatment of ADHD to prevent secondary outcomes could therefore have a positive impact on sexual communication in young adulthood. It could also benefit the development of healthy romantic relationships, since low self-esteem is related to poor relational quality [49], which is illustrated in our study as difficulty trusting a partner and fear of being rejected.

Finally, the women in the study describe misjudging the sexual situation. Trusting the wrong partners, thinking a situation would be safe, could be explained by inattentiveness or inability to perceive risks, previously proposed to make young women with ADHD more vulnerable to exploitation by offenders [24]. Screening for sexual victimization could therefore be important in health care to provide support and prevent re-victimization in young women with ADHD. The issue of expectations of peers that the women in our study describe influence when and with whom they have sex can also be important to raise in health care facilities as peer pressure previously has been shown to result in casual sex associated with sexual assault in young women [50]. The women in our study could possibly be more receptive to others’ opinions, striving to fit into the social context, since girls with ADHD have fewer friends and are more likely to be rejected by peers [51].

A strength of this study is that it is one of the few studies, to our knowledge, that uses a qualitative design to describe aspects of sexual and reproductive health in young women with ADHD [52]. The semi-structured interviews also allowed detailed and varied information about the experiences to be obtained [35]. Even though the sample size is sometimes questioned in qualitative studies, the sample size here was found appropriate for the design, matching descriptions of sample size according to Brinkmann and Kvale [53]. Differences in age and place of recruitment could also have contributed to the richness of data [35].

A limitation of this study may be the use of both a focus group and individual interview, as group interaction can generate a different type of content than individual interviews. However, according to Lambert and Loiselle [54], combining focus groups and individual interviews can enhance the richness of data and broaden understanding of the phenomena in question [54]. There may also be limitations in transferability. The quite large age differences in the sample may make transferability to other groups difficult [40], considering that age can affect experiences. The median age of 25 years may imply that the findings do not reflect the younger women in the sample to the same extent as the older women. However, the life experience of the older women did contribute to better understanding of how self-knowledge and strategies had developed over time and how early experiences affected later experiences. Transferability may also be limited since the sample included women with different types of ADHD, and four women with an additional diagnosis of ASD. However, the heterogenic sample could also reflect the variation of experience despite the type of diagnosis.

Future research investigating young women with ADHD could benefit from studying samples with different types of ADHD separately. Quantitative studies could also help to differentiate sexual behaviors and reasons for sexual victimization that are specifically associated with being a young woman with ADHD compared to a young woman without ADHD. Furthermore, more knowledge is needed to better understand the women’s need for professional support and counseling. According to Murdaugh et al. [34] understanding the women’s motivations for behaviors as perceived benefits and barriers and self-efficacy, as well as interpersonal influences of social norms could help in the development of interventions in health care settings promoting healthy sexual behaviors.

## Conclusions

This study deepens the understanding of how ADHD can affect young women’s emotions and sexual behaviors, thereby leading to challenges in sexual relationships. The results highlight the need for understanding and acceptance by peers and partners because the women feel different from others. The study also contributes knowledge by accentuating the value of involving the partner in counseling, as well as the importance of promoting self-knowledge. Experiences of sexual assault found in the study further emphasize the importance of screening for sexual victimization in professional settings, as well as early diagnosis to prevent secondary outcomes of ADHD.

## Data Availability

The datasets generated and/or analyzed during the current study are not publicly available due to privacy concerns given the limited sample size but are available from the corresponding author on reasonable request and in accordance with the consent and restrictions of the ethical approval.

## Abbreviations

ADHD: Attention Deficit Hyperactivity Disorder
ASD: Autism spectrum disorder
COREQ: Criteria for reporting qualitative research
DSM-5: Diagnostic and statistical manual of mental disorders
STD: Sexual transmitted disease

## Declarations

### Authors’ information

Credentials: KW: RN, RM; IWL: PhD, RN; LH: PhD, RN; SA: PhD, RN, RM; SH: PhD, RN. Occupation at them time of the study: KW: PhD-student, RM; IWL: Senior lecturer; LH: Senior Lecturer; SA: Associated professor; SH: Associated professor.
